# Brain Cortical Area Characterization and Machine Learning-Based Measure of Rasmussen’s S-R-K Model

**DOI:** 10.3390/brainsci15090981

**Published:** 2025-09-12

**Authors:** Daniele Amore, Daniele Germano, Gianluca Di Flumeri, Pietro Aricò, Vincenzo Ronca, Andrea Giorgi, Alessia Vozzi, Rossella Capotorto, Stefano Bonelli, Fabrice Drogoul, Jean-Paul Imbert, Géraud Granger, Fabio Babiloni, Gianluca Borghini

**Affiliations:** 1 Department of Molecular Medicine, Sapienza University of Rome, Piazzale Aldo Moro, 5, 00185 Rome, Italy; amore.2069380@studenti.uniroma1.it (D.A.); daniele.germano@uniroma1.it (D.G.); gianluca.diflumeri@uniroma1.it (G.D.F.); 2 BrainSigns SRL, Via Sesto Celere, 00152 Rome, Italy; pietro.arico@uniroma1.it (P.A.); vincenzo.ronca@uniroma1.it (V.R.); andrea.giorgi@uniroma1.it (A.G.); alessia.vozzi@brainsigns.com (A.V.); rossella.capotorto@uniroma1.it (R.C.); fabio.babiloni@uniroma1.it (F.B.); 3 Department of Computer, Control, and Management Engineering Antonio Ruberti, Sapienza University of Rome, Piazzale Aldo Moro, 5, 00185 Rome, Italy; 4 Department of Anatomical, Histological, Forensic & Orthopedic Sciences, Sapienza University of Rome, Piazzale Aldo Moro, 5, 00185 Rome, Italy; 5 DeepBlue srl, Piazza Buenos Aires 20, 00185 Rome, Italy; stefano.bonelli@dblue.it; 6 EUROCONTROL, Rue de la Fusée 96, 1130 Brussels, Belgium; fabrice.drogoul@eurocontrol.int; 7 École Nationale de l’Aviation Civile, 7 Avenue Edouard Belin, 31000 Toulouse, France; jean-paul.imbert@enac.fr (J.-P.I.); geraud.granger@enac.fr (G.G.); 8 Department of Physiology and Pharmacology “Vittorio Erspamer”, Sapienza University of Rome, Piazzale Aldo Moro, 5, 00185 Rome, Italy; 9 College of Computer Science and Technology, Hangzhou Dianzi University, Hangzhou 310018, China

**Keywords:** cognitive control behaviour, S-R-K model, decision-making, neurophysiological characterization, sLORETA, EEG, brain cortex, Brodmann areas, machine learning, KNN

## Abstract

Background: the Skill, Rule, and Knowledge (S-R-K) model is a framework used to describe and analyze human behaviour and decision-making in complex environments based on the nature of the task and kind of cognitive control required. The S-R-K model is particularly useful in fields like human factor engineering, system design, and safety-critical industries because it helps to understand human errors and how they relate to different levels of cognitive control. However, the S-R-K model is still qualitative and lacks specific and quantifiable metrics for determining what kind of cognitive control a person is using at any given time. This aspect makes difficult to directly measure and compare performance across the three levels. This study aimed therefore to characterize the S-R-K model from a neurophysiological perspective by analyzing the operator’s cerebral cortical activity. Methods: in this study, participants carried out experimental tasks able to replicate the Skill (tracking task), Rule (rule-based navigation) and Knowledge conditions (unfamiliar situations). Results: participants’ Electroencephalogram (EEG) was recorded during tasks execution and then Global Field Power (GFP) was estimated in the different EEG frequency bands. Brodmann areas (BAs) and EEG features were then used to characterize the S-R-K pattern over the cerebral cortex and as inputs to build up the machine learning-based model to estimate participants’ cognitive control behaviours while dealing with tasks. Conclusions: the results demonstrate the possibility of objectively measuring the different S, R and K levels in terms of brain activations. Furthermore, such evidence is consistent with the scientific literature in terms of cognitive functions corresponding to the different levels of cognitive control.

## 1. Introduction

### 1.1. S-R-K Model

The S-R-K model, introduced by Rasmussen in 1983 [1], delineates three cognitive control behaviour modalities. The concepts of Skill, Rule, and Knowledge pertain to the level of conscious control individuals exert during tasks [2]. Specifically, Rasmussen proposed the taxonomy to provide a framework for understanding the wide spectrum of behaviours observed in real-world work environments. This framework extends from the mere observation of information to the execution of physical actions, encompassing various work situations ranging from routine daily tasks to high-stress encounters with unexpected events. Presently, this model finds application in safety-critical human–machine domains, especially in Aviation [3]. In recent decades, numerous endeavors have been undertaken to expand these models to encompass higher-level human decision-making processes, aligning with the escalating levels of automation in operational settings such as aviation, hospitals, and public transport. Additionally, there has been an effort to apply these models to process control applications. It is crucial to acknowledge that humans are not simply predictable input–output machines; rather, they are goal-oriented beings who actively choose their objectives and seek pertinent information to achieve them [1,4].

Building on this foundational framework, the S-R-K model’s practical relevance is particularly evident in safety-critical domains such as air traffic management, healthcare, and industrial process control. Its pragmatic origin, grounded in direct observation of operators in real-world, high-pressure environments, enhances its applicability beyond purely theoretical models. Neurophysiological research has further linked brain activity to different behavioral modes, supporting the development of training programs tailored to operators’ cognitive demands. By applying the S-R-K model, organizations can design targeted strategies to enhance human reliability, reduce incidents related to human factors, and foster a safety culture, even in increasingly automated operational environments.

Skill-based behaviour is characterized by the execution of sensory–motor tasks without the need for conscious control, following the declaration of an intention. These actions unfold smoothly, automatically, and seamlessly. In such tasks, the body operates as a sophisticated continuous control system, adjusting movements based on the surrounding environment. Performance relies on feedforward control and is contingent upon a flexible and efficient internal model of the world. Notably, skilled performance occurs without conscious attention or control, with sensory input unconsciously directed towards elements of the environment essential for updating internal representations. Human activities can be viewed as a sequence of such skilled actions or tasks tailored to specific circumstances. The adaptability of skilled performance arises from the ability to select and combine automated subroutines from a diverse repertoire to achieve objectives [1].

In the Rule-based behaviour, individuals utilize stored rules or procedures, acquired through past experiences or external sources like manuals or operators, to consciously govern the sequential structure of subroutines within familiar work scenarios [1]. Rule-based behaviours are characterized by the application of rules of the form “if <state X> then <action Y>” [5]. At this level, behaviour necessitates the conscious recognition of the situation, followed by the retrieval of relevant rules from pre-existing knowledge and their subsequent application, which can be articulated by the participant. The distinction between Skill-based and Rule-based performance is not always clear-cut, and it often hinges on the individual’s level of training and attentional focus. Typically, Skill-based performance unfolds seamlessly without requiring conscious attention from the individual. Consequently, they may struggle to articulate the specific control mechanisms or informational cues guiding their performance [1].

In unfamiliar situations devoid of prior Knowledge or established control rules, individuals must rely on Knowledge-based behaviour. This cognitive control level involves a behaviour where operators utilize existing knowledge to navigate and solve problems, choosing the most appropriate solution for the given scenario. Following the execution of a response, operators must evaluate available feedback before proceeding further [2]. Successful solutions may be stored as rules for future reference. Overall, processes associated with Knowledge-based behaviour are often slow and cognitively demanding [1].

**Figure 1:**
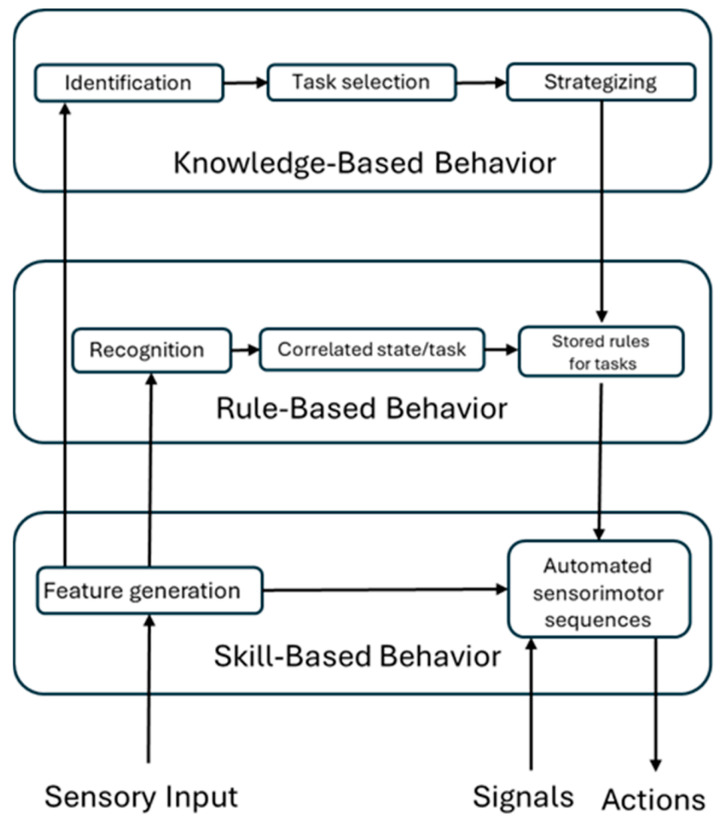
The S-R-K -based model (adapted from Rasmussen 1983 [1]).

In summary, Skill-based behaviour relies on automatic actions, while Rule-based behaviour combines long-term memory retrieval with those automatic actions. Knowledge-based behaviour, in turn, integrates information processing and decision-making with Rule-based procedures and Skill-based automatic actions (Figure 1).

Based on the above descriptions, the present study aimed to provide an objective and quantitative characterization of the three cognitive control levels described in Rasmussen’s Skill-Rule-Knowledge (S-R-K) model by analysing participants’ brain activity and successively applying machine learning techniques to demonstrate the possibility to measure it while dealing with tasks.

Specifically, the study addressed the following research questions:

Can the Skill, Rule, and Knowledge levels be characterised by different brain activation patterns?

Can EEG features be used to classify S-R-K levels using a supervised machine learning model?

Accordingly, we formulated the following hypotheses:

**H1:**  *The three cognitive control levels (Skill, Rule, Knowledge) are associated with distinct brain activation patterns, both in terms of brain regions and frequency bands involved.*

**H2:**  *A supervised classification model can significantly distinguish the participant’s cognitive control behaviour levels.*

While real-world S-R-K applications often involve dynamic, multimodal inputs and stressful conditions, this study standed as the first to comprehensively characterize neurophysiologically Rasmussen’s S-R-K model within a highly controlled laboratory environment. In fact, some of the Authors already demonstrated the possibility to discriminate S-R-K levels in real contexts [3]. However, as described in those studies, potential biases could be due to realistic settings and a more controlled study was needed.

The choice of performing this study under controlled settings enabled the effective isolation of specific neurophysiological correlates for Skill, Rule, and Knowledge levels. Minimizing external confounding factors ensured higher purity and reliability of EEG signals, allowing for robust identification of distinct neural patterns for each cognitive modality. The controlled setting also facilitated the establishment of clearer, more direct relationships between experimental conditions and observed brain responses. This is crucial for building a solid scientific foundation and validating EEG’s ability to discriminate between these qualitatively different cognitive states.

### 1.2. Cognitive S-R-K Level Characterization

A central process that spans all three types of cognitive control behaviours in Rasmussen’s model is the decision-making process. In all three types of behaviours (S, R and K), we assume the involvement of the cingulate cortex, an area that according to [6] is associated with this function. Specifically, at the Skill level, decision-making represents the fundamental core of actions, involving automatic and highly practiced responses. As one moves to the Rule and Knowledge levels, the decision-making process becomes even more prominent, requiring the use of deeper Knowledge and the evaluation of multiple options before acting [1].

The cognitive function of retrieval memory should be particularly prominent in Rule behaviour as it is crucial for recalling relevant rules from pre-existing knowledge. In addition, long-term memory and, particularly, recognition, semantic, and episodic memory are fundamental for this type of behaviour as they allow us to act based on experience. According to [7], multiple lines of investigation provide substantial evidence indicating the involvement of the angular gyrus in the retrieval of both episodic and semantic information. According to [8], functional magnetic resonance imaging (fMRI) research suggests that envisioning specific future experiences (episodic simulation) and recalling specific past experiences (episodic memory) are linked with increased activity in a shared group of brain that includes the hippocampus, medial prefrontal cortex, and left angular gyrus, alongside other regions. In addition, the entorhinal cortex is believed to facilitate the swift formation of new associations by acting as a bridge between the hippocampus and neocortical regions. While the entorhinal–hippocampal interplay is crucial for the initial acquisition of memory, the entorhinal cortex continues to play a role in memory retrieval even after the hippocampus becomes dispensable [9]. It is important to clarify that even in Knowledge-based behaviour, reference is made to past knowledge, thus potentially involving the utilization of long-term memory.

According to Rasmussen [1], in unfamiliar situations, performance control must shift to higher cognitive levels, where behaviour is goal-oriented and Knowledge-based. The action plan is developed through a selection process in which each attempt is tested against the goal, either through trial and error or conceptually predicting the effects of the considered plan. From this perspective, it is crucial to predict the outcome of an action. Over the last ten years, there has been a notable evolution in research concerning the retro splenial cortex. This area of the cortex has risen in significance as a central component of a core network of brain regions supporting various cognitive functions, such as episodic memory, spatial orientation, mental simulation, and prospective planning [10]. Lastly, due to the experimental task being a visuomotor activity, involvement of the occipital area is anticipated in all three types of behaviours. Specifically, involvement of the primary visual cortex, secondary visual cortex, and associative visual cortex is expected across all three types of behaviours (Table 1).

## 2. Materials and Methods

### 2.1. Participants

Eleven healthy male volunteers (26 ± 3 years old) consented to participate in the experiment. The study adhered to the principles outlined in the Declaration of Helsinki of 1975, as revised in 2000. Approval for the research protocol was obtained from the Ethics Committee of Sapienza University of Rome (Prot. CE/PROG.604 dated 5 April 2017). Participation was limited to healthy, normal individuals who volunteered for the study. Each participant provided written informed consent after receiving a thorough explanation of the study. Careful consideration was given to participant selection to ensure sample homogeneity, with recruitment focused exclusively on male participants for the assessment of cognitive control behaviour.

### 2.2. Experimental Tasks

**Figure 2:**
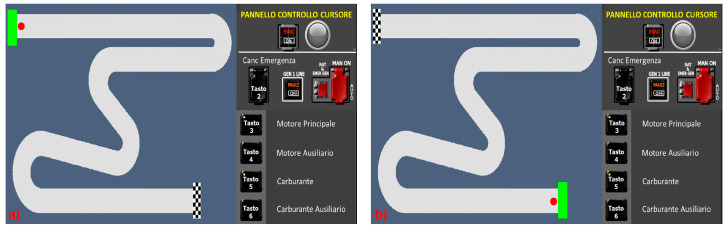
Skill-based task conditions: (**a**) Skill 1—S1, (**b**) Skill 2—S2.

The experimental task and conditions were designed in accordance with Rasmussen’s original S-R-K model description [1]. Specifically, six conditions were designed to elicit Skill, Rule, and Knowledge behaviours with two conditions for each cognitive control level. These conditions were labelled as Skill 1 (S1), Skill 2 (S2), Rule 1 (R1), Rule 2 (R2), Knowledge 1 (K1), and Knowledge 2 (K2). The S1 and S2 conditions (Figure 2) entailed straightforward tracking tasks where participants maneuverer a cursor from the starting point (green box) to the finish line (checkered flag) using a joystick. S1 and S2 tasks required a mere automated sensorimotor sequence.

**Figure 3:**
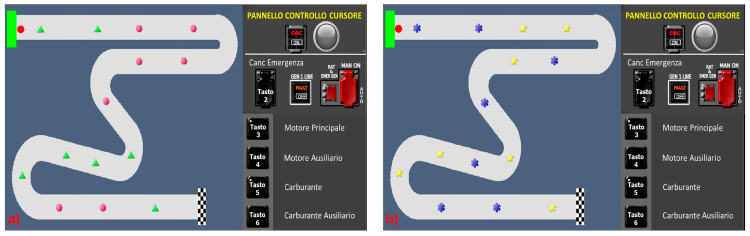
Rule-based task conditions: (**a**) Rule 1—R1, (**b**) Rule 2—R2.

In the R1 and R2 conditions (Figure 3), obstacles were introduced requiring participants to apply specific rules to navigate throughout the path to the arrival. In particular, the obstacles and the corresponding rules to be applied were as follows:

- Triangle 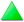: raise the altitude by moving the lever upwards.
- Circle: 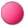: turn right the circle with respect to the moving direction.
- Star: 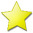: lower the altitude by moving the lever downward.
- Asterix 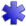: turn left the asterisks with respect to the moving direction.

In the R1 condition, both the triangle and the circle were introduced (Figure 3a), whereas in the R2 condition, only the star and asterisk were added (Figure 3b). This adjustment aimed to enable participants to effortlessly retain the rules. R1 and R2 tasks thus required participants to recognise the condition, correlate it with the rule to be applied, and finally apply the corresponding rule.

**Figure 4:**
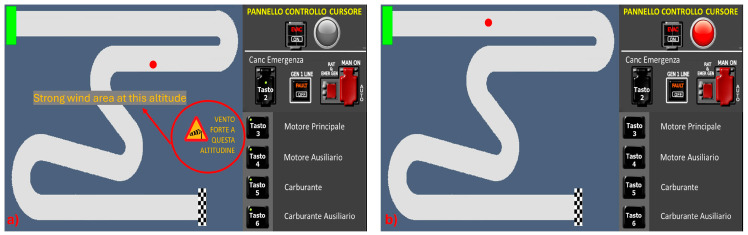
Knowledge-based task conditions: (**a**) Knowledge 1—K1, (**b**) Knowledge 2—K2.

The K1 and K2 conditions were designed to make participants face unfamiliar and challenging situations where prior experiences or knowledge could not offer direct solutions (Figure 4). In the K1 condition, participants encountered a strong wind area upon entering an air sector, causing the cursor to move randomly without any control. When this started, a warning message flashed on the interface, indicating “Strong wind share on this altitude” to offer a hint (Figure 4a). To resolve the situation, participants needed to adjust the altitude level to exit the windy area and then guide the cursor to the finish line figuring out how to control it. Understanding the rotation of the joystick axes by 90 degrees was essential for successful navigation. In the K2 condition, during the initial segment of the track, the cursor suddenly frozen, and the red light on the right panel began flashing (Figure 4b). To regain control of the cursor, participants had to follow a specific sequence representing the following steps:

1. Acknowledge the emergency (press button 2).
2. Notice that the engine and fuel buttons are off, and consequently, activate the auxiliary ones, starting with the fuel (press button 6).
3. Lastly, activate the auxiliary engine (press button 4).

After applying the correct sequence, the emergency light ceased flashing and turned green meaning that the emergency had been solved. Subsequently, the cursor could be taken to the finish line without encountering any further command failures. K1 and K2 tasks required the application of the “Identification, Task Selection, and Strategizing” sequence to solve the ongoing conditions.

### 2.3. Experimental Protocol

The experimental protocol consisted of asking the participants to deal with the different experimental conditions. These six conditions, two for each cognitive control behaviour level, were randomized to mitigate habituation and expectation effects. Prior to the formal experiment, all participants also completed a practice session to mitigate potential learning-related biases. The total duration of the experimental protocol was approximately 30 min.

### 2.4. Behavioural Data

For evaluating participants’ performance, we recorded the time required to complete each experimental condition. Subsequently, distinct accuracy indicators were computed separately for each cognitive control behaviour level. For each *Skill* condition, the participant’s accuracy was compared with the ideal track in terms of length (*L*) and error (*err*) using the following metrics (Equation (1)):

(1) $${Skill}_{\%}=\left(1-\left|\frac{L-L_{opt}}{L}\right|\right)*\left(\frac{{err}_{MAX}}{{err}_{MAX}+err}\right)*100$$

where

*L_opt_*: length of the ideal track;

*L*: length of the track completed by the participant;

*err_MAX_*: maximum distance of the cursor from the ideal track during the entire run

*err*: average of these distance values for all runs.

In the *Rule* condition, a comparison was made between the number of successfully applied rules and the total number of rules to be applied as follows:

(2) $${Rule}_{\%}=\left(\frac{\sum_{1}^{7}turn+\;\sum_{1}^{7}w*alt}{14}\right)*100$$

The equation compares the number of successfully applied rules to the total number of Rules for each R1 and R2 condition. The denominator, 14, represents the total number of rules for each condition. The parameters *turn* and *alt* can take values of 1 or 0 depending on whether the corresponding rule (to turn or change altitude) has been executed correctly or not, respectively. Additionally, the *alt* parameter is weighted by a factor *w*, which accounts for instances where the participant maintains the cursor up or down continuously for consecutive targets.

In the K1 condition, the comparison was made between the ideal time necessary to complete the circuit and the time required by the participant, using the following equation:

(3) $${Knowledge1}_{\%}=\;\left(\frac{T_{opt}}{T_{cir}+T_{ev}}\right)*100$$

The equation compares the ideal time necessary to complete the circuit *T_opt_* with the time spent by the participant to complete the event (increasing or decreasing the altitude) T_ev_ and the time spent by the participant to complete the whole circuit (*T_cir_*). The accuracy for the *Knowledge*2 condition was evaluated by giving equal weight to two parameters: the number of attempts (*n_att_*) made to reach the correct button sequence, and the percentage of time used (*T*) relative to the available time to solve the failure (*T_av_* set to 20 min), using the following equation:

(4) $${Knowledge2}_{\%}=\left(\frac{1}{n_{att}}\right)*50+\;\left(\frac{T_{av}-T}{T_{av}}\right)*50$$

To establish a level of accuracy relating to the performance of the experimental task, each of these metrics was converted into percentages. This enabled the aggregation of performance across the different experimental conditions. For instance, the average accuracy of Skills was computed by combining the accuracy of S1 and S2, and the same approach was applied to the other conditions (Rule and Knowledge).

### 2.5. Subjective Data

An ad hoc questionnaire was created to evaluate how participants perceived their performance in terms of cognitive control behaviour. The questionnaire was developed based on Rasmussen’s three levels of cognitive control behaviour, focusing on aspects where participants could provide ratings:

- Level of attention;
- Task automation level;
- Familiarity with the task.

Participants completed this questionnaire at the end of each experimental condition and an average score was estimated for cognitive control level and then compared statistically.

### 2.6. EEG Data Recording and Analysis

**Figure 5:**
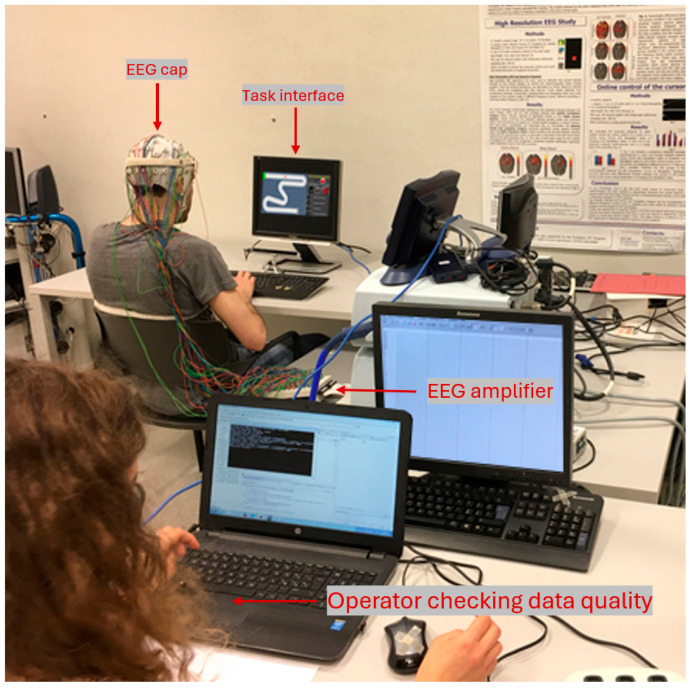
The experimental setup for EEG recording is depicted. Electrodes were placed on the scalp according to the International 10-10 system to capture brain activity. The EEG signals were recorded through a digital high-resolution EEG system. The participants were seated comfortably in a controlled environment, with minimized external stimuli to reduce noise in the data.

Throughout the experiment, the electroencephalogram (EEG) and electrooculogram (EOG, utilized solely to eliminate eye-related artifacts from EEG data) signals were recorded using a high-resolution (BrainAmp, Brain Products, Gilching, Germany) 61-channel system (Fp_1_, Fp_z_, Fp_2_, AF_7_, AF_3_, AF_z_, AF_4_, AF_8_, F_7_, F_5_, F_3_, F_1_, F_z_, F_2_, F_4_, F_6_, F_8_, FT_7_, FC_5_, FC_3_, FC_1_, FC_z_, FC_2_, FC_4_, FC_6_, FT_8_, T_7_, C_5_, C_3_, C_1_, C_z_, C_2_, C_4_, C_6_, T_8_, TP_7_, CP_5_, CP_3_, CP_1_, CP_z_, CP_2_, CP_4_, CP_6_, TP_8_, P_7_, P_5_, P_3_, P_1_, P_z_, P_2_, P_4_, P_6_, P_8_, PO_7_, PO_3_, PO_z_, PO_4_, PO_8_, O_1_, O_z_, O_2_) with a sampling frequency of 250 (Hz). Participants were seated 60 cm away from the monitor (Figure 5). Before starting the experimental tasks, a one-minute baseline period of data collection was conducted. During this baseline period, participants were instructed to observe the cursor moving toward the practice track without any active participation (REF condition).

All EEG electrodes were referenced to both earlobes and grounded to the left mastoid, with electrode impedances maintained below 10 kΩ. Bipolar electrodes for the EOG were positioned vertically above the left eye. The EEG signal was first band-pass filtered with a 5th-order Butterworth filter in the interval 2–45 (Hz). The eye blink artefacts were detected and corrected online by employing the o-CLEAN method [11]. For further sources of artefacts, specific algorithms of the EEGLAB toolbox [12] were applied. Specifically, the pre-processed EEG signal has been divided into 1 s long epochs. A Threshold criterion has been applied to recognize artifactual data automatically. EEG epochs with signal amplitudes exceeding ±80 μV were marked as “artifacts”. In the end, EEG epochs marked as “artifacts” were removed from the EEG dataset with the aim of having a clean EEG signal to perform the analyses. The average number of rejected epochs due to artifacts was very low (0.36 ± 0.92 epochs, corresponding to 0.03 ± 0.08%), indicating high data quality and an effective artifact rejection procedure.

From the artifact-free EEG, the Global Field Power (GFP) was calculated for the EEG frequency bands using a Hanning window of 1 s (which means 1 Hz of frequency resolution). The EEG frequency bands (Delta, Theta, Alpha High, Beta1, Beta2, Beta3, and Gamma) were defined according to the Individual Alpha Frequency (IAF) value [13] computed for each participant. Since the Alpha peak is mainly prominent during rest conditions, the participants were asked to keep their eyes closed for one minute before starting the experiment. Such a condition was then used to estimate the IAF value specifically for each team member.

The Brodmann reference model [14] was then used to identify the cortical areas and corresponding cognitive functions significantly activated under the Skill, Rule, and Knowledge conditions. The cortical area analysis was conducted using a 66 (Brodmann areas—BAs) × 6 (frequency bands) × 11 (participants) matrix. The coordinates of the BAs were derived from [15,16]. A key step was the resolution of the inverse problem, which involves identifying neural sources from surface EEG signals [17]. This provides an accurate map of underlying brain cortical activity. The estimation of the current density strength for each dipole was achieved by solving the electromagnetic linear inverse problem [18,19]. Specifically, the solution to the linear system

A**x** = b + n (5)

at a specific time *t* provides an estimate of the dipole source configuration *x* that produces the measured EEG potential distribution *b* at that same time. The model also incorporates measurement noise *n*, which is assumed to be normally distributed. The matrix *A* represents the lead field matrix, with each *j*-th column indicating the potential distribution created on the scalp electrodes by the *j*-th unit dipole [20].

Data were normalized using the z-score formula. For each participant, each condition, and each of the 66 BAs, the mean and standard deviation over time were calculated. The z-score was computed by subtracting the mean and dividing by the standard deviation for each second of data, using the REF condition as a reference. This reduced individual differences, allowing for a more accurate comparison of brain activity across participants and experimental conditions. Statistical analyses were performed separately for each EEG band and cortical region, rather than across all bands, regions, and time points, to avoid multiple comparison issues. For each experimental condition and brain area, a vector was created for each participant, and a Kolmogorov–Smirnov normality test was applied. If the data were normally distributed, a Student’s *t*-test was used, otherwise Kruskal–Wallis test was used to identify brain areas which did differ among the Skill, Rule and Knowledge conditions. This process generated 66 ROIs × 6 EEG-bands matrices for cortical areas, representing neural activity across experimental conditions. In addition, we selected only those cortical areas commonly activated between the two repetitions of each cognitive control behaviour level (S1 + S2 = S; R1 + R2 = R; K1 + K2 = K) to avoid those linked to potential familiarization or learning phenomena and reduce potential false positives. 

The cortical data were graphically represented using the sLORETA software (version 2008-November-04) [21]. EEG cortical data of each participant were estimated and localized in the whole grey matter volume by applying the source localization algorithm standardized LOw-REsolution brain electromagnetic Tomography (sLORETA) [21] implemented in sLORETA software [22,23]. This approach allowed to estimate the current density distribution of cortical and subcortical brain sources from the scalp electric potentials with zero localization error. As a solution of the forward model reproducing the electromagnetic propagation from the active sources to the EEG sensors, we used a lead field matrix obtained from the application of the Boundary Element Method [24] to the MNI152 realistic head model [25]. We used a 3D lead field matrix modelling the propagation of 6239 active sources distributed in the whole grey matter at 5 mm spatial resolution towards the EEG sensors. The regularization parameter λ used for sLORETA solution was computed by means of a cross-validation approach [26]. The solution of the source localization problem for each participant and experimental condition consisted of 6239 waveforms (one for each dipole) used to model the grey matter. Since we wanted to characterize the S, R and K levels considering only pure cognitive functions, the Fp, FC, C, CP, T, and TP EEG channels were excluded from the analyses as already did in previous studies [20].

The process of channels selection followed the same methodological approach as the selection of ROIs. In both cases, statistical analyses were conducted to identify those brain areas significantly activated with respect to the REF condition. While the selection of ROIs pertains to a specific set of brain regions, the scalp data analysis involves 61 channels. Despite this difference in scale, the procedure remains consistent, ensuring a rigorous and comparable approach.

### 2.7. Machine Learning-Based SRK Index

The study explored the use of a supervised machine learning model to classify the cognitive control behaviour levels (S, R and K). In particular, given the novelty of this study, there were no documented models in the literature that could be used for this purpose. Some of the authors of this study previously demonstrated the possibility to discriminate the S, R and K levels [3,27], but the work was performed in realistic settings, hence with some limitations. On the contrary, this study aimed at characterising the S, R and K levels under controlled settings to avoid biases due to uncontrolled variables.

To identify the most suitable approach for this classification task, we tested several supervised machine learning algorithms, including Decision Tree (DT), Random Forest (RF), K-Nearest Neighbour’s (KNN), and Gradient Boosting (XGBoost). All analyses presented were conducted in Python (version 3.11) using the Scikit-learn library [28].

The choice of algorithms and hyperparameter ranges was not arbitrary. We adopted the set of models and tuning grids implemented in the Mindtooth tool, which is specifically designed for EEG data analysis. This approach ensured methodological consistency with established practices rather than relying on an exploratory model search. Similar models and parameter grids have also been employed in previous EEG classification studies [29,30], further supporting the validity of this choice.

Decision Tree (DT): The Decision Tree algorithm creates a hierarchical structure of conditional nodes to classify observations. Each internal node represents a decision based on feature values, while terminal nodes provide the final classification. This method offers high interpretability, making it valuable for understanding which features are most important in distinguishing between cognitive control levels. However, individual decision trees can be prone to overfitting, especially with limited data (Table 2).

Random Forest (RF): Random Forest is an ensemble method that combines multiple decision trees to improve classification performance. Each tree is trained on a random subset of features and data, and the final classification is determined by majority voting among all trees. This approach reduces overfitting compared to individual decision trees and provides better generalization, while still maintaining reasonable interpretability through feature importance rankings (Table 3).

### 2.8. Random Forest Easy (RF-Easy)

We developed a lighter version of the Random Forest model, in which only the number of estimators and the maximum tree depth were optimized—using the same values as in the previous model. This approach was aimed at reducing computational cost during training.

K-Nearest Neighbors (KNN): KNN is a non-parametric algorithm that classifies observations based on the majority class of their k nearest neighbors in the feature space. It is considered a “lazy learner” because it requires no explicit training phase, simply storing all training instances. The algorithm is particularly suitable for datasets with limited records and high-dimensional feature spaces, as it does not make assumptions about the underlying data distribution (Table 4).

Gradient Boosting (XGBoost): XGBoost employs an iterative boosting approach where weak learners (typically decision trees) are sequentially added to correct the errors of previous models. The algorithm uses gradient optimization to minimize classification errors at each step while incorporating regularization techniques to prevent overfitting. This method is particularly effective for complex pattern recognition tasks and can handle high-dimensional data well (Table 5).

Given the large number of features (61 EEG channels × 6 EEG frequency bands) compared to the limited number of records per session (30–256 records), we reduced the dataset size by applying Principal Component Analysis (PCA) [31]. Since it was not possible to select a fixed number of principal components that would be suitable for each subject, we decided to select a different number of principal components for each session based on the amount of variance explained by the data. The method trained on the dataset (session) was then used to transform both training and testing datasets before running the classification model.

The accuracy of the models was evaluated using a confusion matrix, which provided a basis for calculating key metrics such as overall accuracy, specificity, recall, precision, and the F1-score. Specifically, the analysis considered the effect of overlapping windows of variable sizes, calculating the mode for each window to identify trends in the models’ performance.

The machine learning classifier provides an objective and quantitative tool to distinguish between Skill, Rule and Knowledge levels of cognitive control based on EEG data. While sLORETA representations enable the visualization of cortical activations associated with each cognitive state, they require expert interpretation and do not offer a direct method for classification. In contrast, the supervised classifier translates complex neurophysiological patterns into measurable outcomes, allowing for automatic identification of higher-order cognitive engagement (Knowledge-based behavior). This approach reduces the subjectivity inherent in visual inspection and supports scalability, cross-subject generalization, and the potential for real-time monitoring of cognitive control strategies in applied or operational contexts.

## 3. Results

### 3.1. Behavioural Data Results

**Figure 6:**
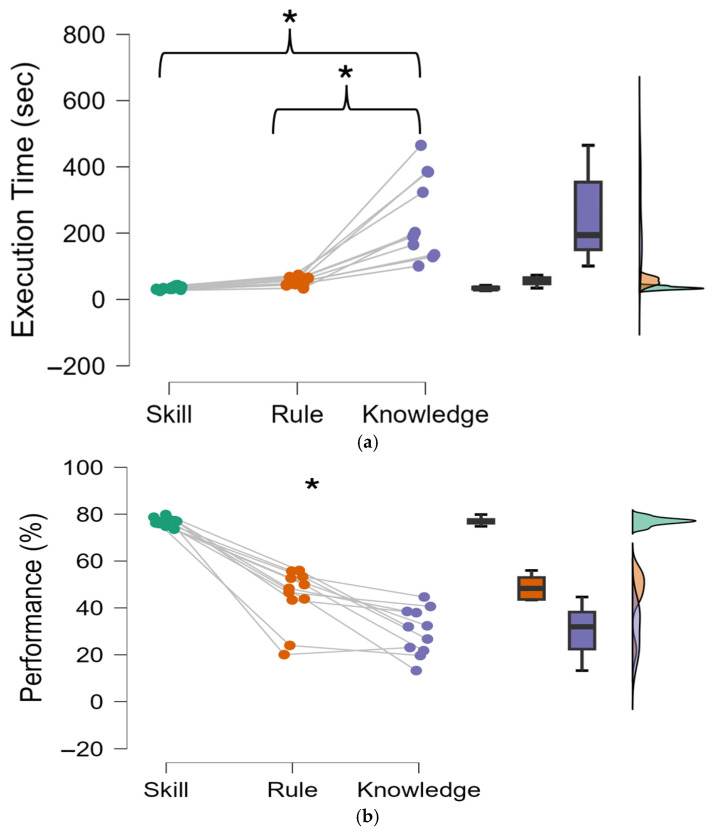
Difference in Completion time (**a**) and accuracy (**b**) for different levels in cognitive control tasks. Asterisks represent conditions reporting statistical differences (*p* < 0.05). Green indicates the Skill condition, orange indicates the Rule condition, and purple indicates the Knowledge condition.

A repeated-measures ANOVA was performed on average values of performance achieved during the S-R-K levels. If the ANOVA returned significant differences (*p* ≤ 0.05), a Bonferroni post hoc test was conducted. Figure 6a depicts a noteworthy increase in the time taken to complete the circuit under the Knowledge condition compared to both Skill (*p* < 0.001, Cohen’s d = −2.89, CI = −314.63 ÷ −102.26) and Rule conditions (*p* = 0.001, Cohen’s d = −2.61, CI = −291.86 ÷ −83.94). Figure 6b illustrates a significant decrease in the task accuracy (performance) from the Skill (*p* < 0.001, Cohen’s d = 5.15, CI = 38.3 ÷ 55.41) and Rule (*p* < 0.006, Cohen’s d = 1.63, CI = 4.59 ÷ 25.08) with respect to the Knowledge condition.

### 3.2. Subjective Data Results

The Subjective data was collected via questionnaires. We considered the items pertinent to the various cognitive control conditions (Skill, Rule, and Knowledge) and averaged them across the two repetitions. Subsequently, a repeated-measures ANOVA was conducted on the corresponding scores. The results (Figure 6) did not show any significant effect across the experimental conditions (F(2, 20) = 2.3; *p* = 0.12). However, they reported a more “unfamiliar” perception and needs for “reasoning and planning” in the Knowledge conditions than the Skill ones.

### 3.3. Neurophysiological Data Results

Table 6 highlights the cortical activations, in terms of BAs, for each cognitive control behaviour and EEG frequency band. This information is crucial for understanding how different cognitive control behaviours translate into specific brain activation pattern, thereby providing a detailed map of brain activity in response to varying tasks. This result is supported by Rasmussen’s model definition (Figure 1), which highlights that there are common areas involved in all three types of behaviours, as well as specific areas that help to distinguish them.

The results of this analysis have been reported graphically through sLORETA. The images (Appendix A) display 4 orthogonal slices of the brain cortex. Each figure presents four perspectives: the right hemisphere viewed from the right, from above, from the left, and a perspective from below. Brain anatomy is depicted in grey. Brain activity increments with respect to the REF are in red, no activations in white, and brain activity decrements in blue colours. Coloured areas represent the t-values, or the differences in the medians. Over each image, there are letters following the following legend: A = anterior, P = posterior, S = superior I = inferior, L = left, R = right.

### 3.4. Machine Learning-Based SRK Results

The proposed study was designed as an intra-subject classification task aiming to assess whether EEG features allow reliable discrimination of cognitive control levels (e.g., S, R and K) for each individual participant. Accordingly, for each participant two “S-R vs. K” session pairs were recorded: one pair was used for training and hyperparameter tuning, while the other pair was held out for the final testing and classification accuracy evaluation. This session-level separation ensured that no overlapping windows could occur between training and testing sets, thereby preventing information leakage. Moreover, it should be noted that EEG signals are inherently temporally correlated and can be considered quasi-stationary over short periods [32].

### 3.5. 3-Class Classification Results

In our initial analyses, we aimed at classifying the three cognitive control levels (Skill, Rule, and Knowledge). However, this approach did not yield satisfactory classification performance (all accuracy < 0.6), suggesting limited discriminability between the three conditions, particularly between Skill and Rule. For this reason, based on the definition reported in Rasmussen’s model of the S, R and K cognitive controls, and the similarity of brain activation patterns (both in terms of brain region activation and spectral features) of S and R, we have adjusted the classification strategy.

### 3.6. 2-Class Classification Results

Based on this observation, we merged Skill and Rule into a single class (S + R), reflecting lower levels of cognitive control, and contrasted it with the Knowledge (K) class, associated with more reflective processes. This revised binary classification yielded better accuracy. Our results support the idea that cognitive control can be divided into more reflexive, automatic processes and more effortful, controlled strategies

**Figure 7:**
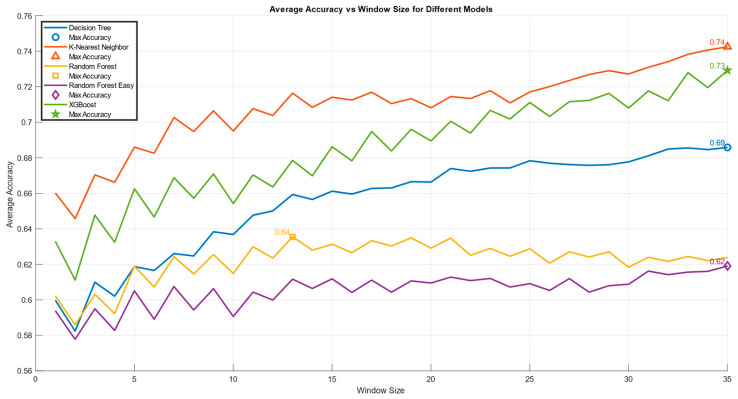
The figure represents the study of the binary condition where “S” and “R” are combined, providing insights into their collective impact on model accuracy. It illustrates the trend of the accuracy metric across all five analyzed models: Decision Tree, Random Forest, Random Forest Easy, K-Nearest Neighbors, and Gradient Boosting. Windows ranging from 1 to 35 were analyzed. The legend indicates the color corresponding to each model and the symbol that marks the point where the accuracy reaches its maximum. The y-axis represents the accuracy, while the x-axis displays the varying window sizes considered in the analysis. This setup allows for a clear comparison of the models’ performance in relation to the size of the windows used.

Figure 7 illustrates the classification accuracy across all five models: Decision Tree (DT), Random Forest (RF), Random Forest Easy (RF-Easy), K-Nearest Neighbors (KNN), and Gradient Boosting (XGB) of the binary condition (PCA90, S.I. 10-10, *p* < 0.05). This graph was generated using a MATLAB (Version: R2022b) script, which facilitated a comprehensive analysis of the accuracy trends as the size of the overlapping windows varying from 1 to 35 for each model. By examining different window sizes, insights can be gained into how the accuracy of each model fluctuates under various conditions, providing a better understanding of their performance and reliability. This analysis emphasizes the significance of window size in evaluating model accuracy and its impact on the overall effectiveness of the predictive models. The results showed how the KNN model returned the best classification accuracy.

**Figure 8:**
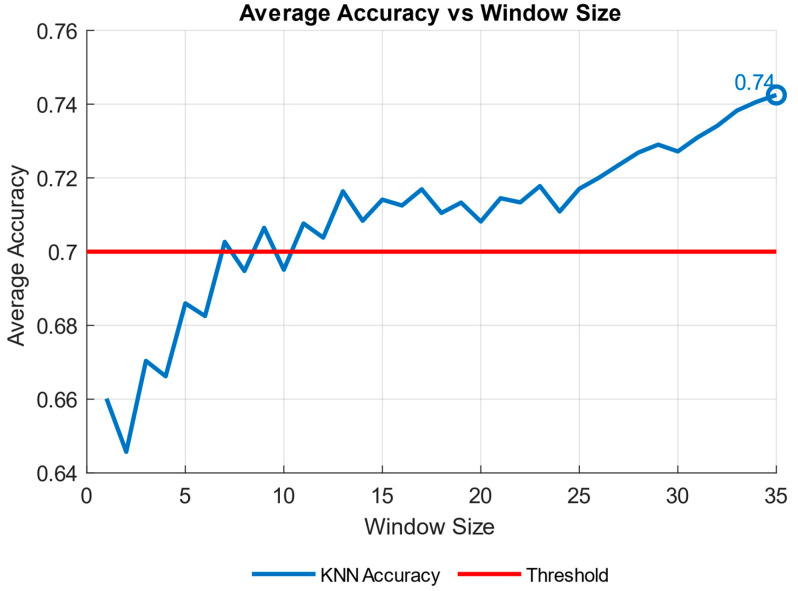
The figure illustrates the trend of the average accuracy of the K-Nearest Neighbors (KNN) model as a function of the window size, ranging from 1 to 35 s. This analysis is conducted within a binary classification framework where conditions “S” and “R” are combined. The y-axis represents the average accuracy, while the x-axis denotes the varying window sizes used in the evaluation. The blue curve depicts the accuracy trend, highlighting the influence of window size on model performance. The red circle marks the first occurrence where the accuracy surpasses the 0.70 threshold, providing a critical reference point for assessing the model’s reliability. This investigation underscores the importance of selecting an appropriate window size to optimize classification performance in predictive modelling.

We therefore investigated different window sizes (from 1 to 35 s) to evaluate the accuracy of KNN in discriminating between the (S, R) and K level (Figure 8).

**Figure 9:**
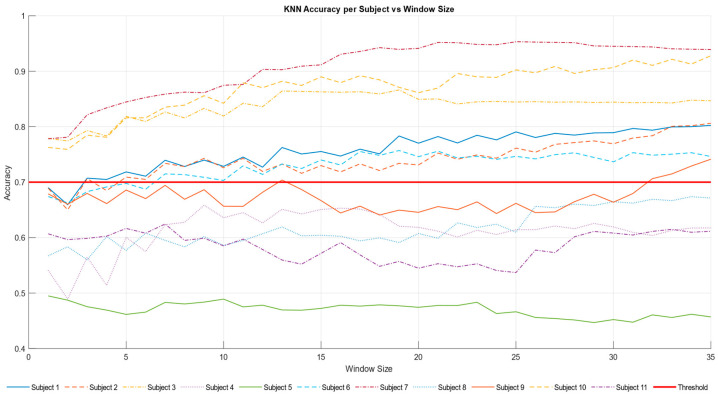
The graph displays the accuracy of the KNN model for each subject in relation to varying window sizes. Each line represents a different subject, using distinct colors and line styles for differentiation. As the window size increases, the accuracy of most subjects tends to rise, indicating improved model performance with larger windows. Notably, seven out of eleven subjects surpass the threshold of 0.70, highlighting the effectiveness of the model. The horizontal red line represents this defined accuracy threshold, allowing for easy comparison across subjects, and is set at 0.70.

From Figure 9 it is possible to see how the model’s accuracy fluctuates for each subject with different window sizes. This provides insights into how the KNN model performed over various time frames and showed how participants responded to changes in the window length.

Based on this analysis, we were able to reach the highest classification accuracy by selecting a window size of 35 s. In Table 7, participants are highlighted in green colour when the corresponding KNN model returns a classification accuracy above 0.70. This visualization clearly demonstrated that many participants achieved high performance, emphasizing the overall effectiveness of the KNN model in this context.

The main metrics for the binary condition are summarized, with a specific focus on window 35, where the highest accuracy was achieved. This highlights the critical role of window selection in optimizing model performance and emphasizes the significance of these metrics in evaluating the effectiveness of the KNN model within this context (Table 8).

## 4. Discussion

The subjective measures did not show any significant differences among the S, R, and K conditions. This lack of differentiation may be due to the limited sensitivity of the questionnaire items, participants’ difficulty in introspectively assessing their cognitive strategies, or the relatively short duration of exposure to each condition, which might not have been sufficient to elicit clearly distinguishable subjective experiences.

Interestingly, behavioural data revealed significant performance reductions under knowledge-based conditions (Figure 6). As the level of cognitive control behaviour increased, moving from Skill to Rule, and finally to Knowledge, the performance decreased as well (Figure 6).

The first hypothesis (H1) posited that the three cognitive control levels—Skill, Rule, and Knowledge—would be associated with distinct patterns of brain activation, both in terms of the specific brain regions engaged and the frequency bands involved.

The analysis of EEG data revealed an increasing number of brain areas engaged as participants progressed from Skill-based to Knowledge-based behavior. This finding aligns well with Rasmussen’s model (Figure 1), which posits that Skill-based behavior involves minimal cognitive effort, while Rule- and Knowledge-based levels require progressively greater cognitive engagement and activation of more cortical regions (Appendix A). This consistency supports a coherent neurophysiological characterization of Skill, Rule, and Knowledge levels in terms of brain areas (BAs) and their related cognitive functions.

Specifically, the Skill-based level is characterized by combined involvement in visual processing, decision-making, and attention. Visual processing engaged the entire beta frequency band in BAs 17, 18, and 19. Previous studies [33] show that beta activity increases during visual attention, suggesting the visual cortex contributes to both basic processing and higher cognitive functions like attention and awareness. Gamma-band activity also involves these areas during Skill-based behavior. According to [34], Gamma synchronization in the visual cortex is linked to attentional stimulus selection. BA 37, corresponding to the fusiform gyrus, was similarly active in the Gamma band during Skill behavior, reflecting enhanced neural responses to visual stimuli driven by attentional mechanisms [35]. The fusiform gyrus also participates in the beta band, supporting advanced visual processing such as object identification [36].

In terms of attention, a high alpha band desynchronization was found in BA 8 (frontal eye field, FEF). The dorsal frontoparietal network, including FEF and intraparietal sulcus (IPS), manages spatial attention distribution, partly through alpha rhythm desynchronization that prepares the brain for visual targets [37]. Decision-making functions involved BA 23 and 24 in the high alpha band. Recent computational models in social neuroscience have highlighted these areas’ roles beyond traditional ‘social brain’ regions, particularly in acquiring self-knowledge during interactions [38]. Memory retrieval, critical for Skill-based behavior through self-learning, was associated with beta oscillations in the ACC (BA 23-24), which predict reward-related bias and optimize automatic responses [36]. BA 30 in the cingulate gyrus was active in beta and Beta 3 bands, while BA 31 showed high alpha desynchronization linked to episodic memory retrieval [39]. Gamma-band activation in BA 23 (ventral posterior cingulate cortex) and BA 30 (angular gyrus) also supports the role of memory retrieval in Skill-based behavior [40]. Coordination between BA 8 and 24 (ACC), reflected by Theta and beta synchronization, further suggests ACC’s key role in cognitive control during tasks involving saccades [41].

Rule-based behavior engages cognitive functions including decision making, memory retrieval, complex processing, and visual attention. Like Skill behavior, beta activity spans BAs 17, 18, and 19 in the visual cortex [33], with Gamma-band activity also present. Theta band activation in BAs 17 and 18 aligns with saccadic rhythms observed during natural scene observation [42]. High-frequency activation of BA 37 again underscores fusiform gyrus involvement, enhancing stimulus-driven Gamma oscillations and visual attention [35]. BA 21 in the Gamma band supports motion perception within the visual field [43]. Decision-making-related Theta activation occurs in BAs 23 and 24, with additional involvement of BAs 30, 31, and 32, reflecting increased planning time and cognitive demands in Rule and Knowledge tasks [44]. Beta and high alpha bands also implicate ACC-related regions (BAs 23-24, 32) in reward monitoring and adaptive behavior [36,38]. Memory-related activations in BAs 23, 30, and 31 reinforce the importance of memory retrieval in applying learned Rules [39,40]. Beta bands engage BA 36 (perirhinal cortex), critical for recognition memory [45], and BA 39, also involved in complex cognitive scenarios [46]. Gamma band involvement of BA 32 highlights its role in target detection, response inhibition, and performance monitoring [47]. Synchronization between BA 8 (FEF) and 24 (ACC) in the Theta band, noted during Skill tasks, similarly supports cognitive control during Rule-based behaviors [41].

Knowledge-based behavior encompasses a broader set of cognitive functions including visual processing, decision making, memory retrieval, problem-solving, and complex cognitive operations. Theta band activation in BAs 17, 18, and 19 supports saccadic visual attention [42], and Gamma activity within these areas emphasizes attentional stimulus selection [34]. BA 21, involved in motion perception, participates in both Rule and Knowledge behavior [43]. The beta band consistently engages visual areas (BAs 17–19) across all behavior types, underlying visual attention’s central role [33]. Fusiform gyrus (BA 37) activation in Gamma band remains consistent across Skill, Rule, and Knowledge behaviors, facilitating advanced visual processing and attentional modulation [35]. BA 39 (Wernicke’s area) also shows activity in Beta 3, Theta, and Gamma bands, reflecting language comprehension and speech production involvement during tasks [48]. Additional areas unique to Knowledge behavior include BA 41 (auditory cortex, Beta 3) and BA 44 (Broca’s area, Beta 3), as well as BA 47 (pars orbitalis) in the alpha band, related to social communication and facial expression. Decision-making in Knowledge behavior specifically involves BA 13 (insula) in the Theta band, a region linked to emotional processing, risk assessment, self-awareness, and complex social functions like empathy [49]. BA 21’s Theta activation highlights its role within the Default Mode Network (DMN) for self-referential thought and memory retrieval during complex decision-making [50]. Consistent involvement of cingulate regions (BAs 23, 24, 30, 31, 32) in Theta and Gamma bands reflects their role in plan elaboration and cognitive control under higher cognitive demands [44]. BA 36 again contributes to recognition memory and contextual evaluation critical for Knowledge behavior [45]. High alpha activity in BA 24 supports social cue processing relevant for informed decision-making [38]. Problem-solving engages BA 10 (anterior prefrontal cortex) in Beta and Gamma bands, a region essential for abstract thinking and strategic planning. DLPFC areas (BAs 9 and 46) activate in alpha bands, mediating cognitive control and emotional regulation during complex tasks [51]. Finally, Theta synchronization between BA 8 (FEF) and 24 (ACC) persists across all behavior types, reinforcing its importance for cognitive coordination during saccadic tasks [41].

The observed patterns of activation across multiple BAs suggest coordinated engagement of large-scale functional networks rather than isolated regional activity. Interactions between frontal, cingulate, and parietal areas are consistent with the recruitment of the Frontoparietal Control Network, while activity in BA 21, BA 39, and cingulate regions aligns with the Default Mode Network. These findings indicate that Skill, Rule, and Knowledge-based behaviors involve not only specific cortical regions but also the dynamic interplay of distributed networks, supporting the view that cognitive control emerges from large-scale functional connectivity rather than single-region activations [52].

The second hypothesis (H2) stated that a supervised classification model would be able to significantly distinguish between the different levels of cognitive control behavior.

Among all the analysed conditions, the one that produced the best results was the binary condition. In this condition, the Skill and Rule behaviours were merged and compared to the Knowledge level. This approach yielded the most promising results (Figure 7), as the tasks performed by participants in the Skill and Rule conditions were highly similar, both from a practical perspective and according to the existing literature, which identifies these two conditions as the most comparable.

The accuracy trend of the KNN model was plotted against the varying time window (Figure 8). The time window represents the duration used to collect data, and the goal was to identify a configuration capable of achieving good results even with reduced windows, indicating greater efficiency. According to the literature [53,54], an accuracy value is considered good when it exceeds 0.70. This threshold was surpassed after 7 s, and a positive trend in accuracy was observed over time. The results (Figure 9, Table 7) show that 7 out of 11 participants surpassed the accuracy threshold. Table 8 underscores the robustness of the KNN model for S-R-K classification using a window size of 35 sec. The per-subject performance metrics for the binary classification (Knowledge vs. Skill + Rule), including Precision, Recall, Specificity, F1-score, and Accuracy for each class, reported as mean ± 95% confidence interval across cross-validation folds, as well as confusion matrix entries (TN, FP, FN, TP), can be found in Table A1 in Appendix A.

Despite the innovative results, it is important to note some limitations of the study. Firstly, the sample size of the experimental group was relatively small. The next step will aim therefore at enlarging the number of participants. Secondly, the characterization and measure of Skill, Rule and Knowledge levels were performed after the execution of tasks, not while dealing with tasks. Thirdly, the machine learning model used, despite its simplicity, demonstrated a moderate level of accuracy in class classification, even in the presence of class imbalance (Table 8, average F1-score = 0.72). However, it is important to acknowledge that the imbalance among classes S, R, and K—especially for class K—may have affected the model’s reliability and should be carefully addressed in future research. Additionally, the results indicate that for some participants, the model did not reach an adequate level of accuracy. This may be due to challenges in feature selection and, more broadly, to the difficulty of designing a model that effectively utilizes a limited set of features given the dataset’s characteristics. Since the length of the runs cannot be determined a priori—being dependent on the time each participant takes to complete the task—it was not possible to predefine a representative set of features for the phenomenon. Future studies could explore two possible directions: (1) analyzing the dataset collected from existing subjects to identify common feature patterns across participants and (2) implementing an ensemble classification model trained on feature subsets, with the final prediction obtained by combining their outputs.

Based on our results, we may assume a similarity between the SRK model and the Kahneman’s Systems 1 and 2 model [55]. In fact, we can assume that Skill and Rule in the SRK model can be associated with System 1, which is fast, automatic, and intuitive. These behaviours are in fact quick and require minimal effort, relying on learned patterns and predefined rules. They enable efficient task handling without deep reflection. On the other hand, Knowledge can be associated with System 2, which is slower, reflective, and rational. It requires a deeper understanding, analysis, and problem-solving, involving conscious, deliberate thinking for more complex tasks [56].

## 5. Limitations

A limitation of the present study is the composition of the sample, which included only young healthy male participants. This homogeneity reduces the generalizability of the findings to populations of different ages or to females. Although no established evidence suggests a specific influence of age or sex on the correlation investigated here, we cannot exclude their potential role. Therefore, the results should be interpreted as applying to this specific population. At the same time, the homogeneity of the sample helped minimize variability from potential confounding factors, thus strengthening the internal validity of the observed associations. Future studies with larger and more diverse cohorts will be necessary to confirm and extend the generalizability of these findings.

## 6. Conclusions

The findings provide strong support for our initial hypotheses. In line with H1, we identified distinct brain activation patterns corresponding to different levels of cognitive control, both in terms of the brain regions and the frequency bands. Concerning H2, the supervised classification model returned a tool by which we could monitor users while performing tasks and assess whether they are dealing with them at the S, R or K level. This capability could play a key role in personalizing training programs (Adaptive Training), user interfaces, optimizing decision support systems, and enhancing Human–Machine interactions.

In summary, this work lays the groundwork for future research and real-world applications of the S-R-K model across a variety of domains.

## Figures and Tables

**Table 1:** The following table summarizes the executive functions involved in skill-based, rule-based, and knowledge-based behaviours. An “X” indicates that the function is involved in that behaviour.

Executive Function	Skill (S)	Rule (R)	Knowledge (K)
Long-term memory		X	X
Mental and cognitive workloads		X	X
Memory retrieval		X	X
Complex and demanding cognitive processing		X	X
Decision Making	X	X	X
Problem solving			X
Mental Simulation			X
Automatic processing	X		
Visual processing	X	X	X

**Table 2:** The table lists the main parameters for configuring a decision tree, including criterion, max depth, mins samples split, min samples leaf, max leaf nodes, max features, and min impurity decrease. These parameters affect the quality of splits, the complexity of the tree, and its ability to generalize.

Parameter	Values
Criterion	gini, entropy
Max depth	from 2 to 100
Min samples split	from 5 to 300
Min samples leaf	from 5 to 200
Max leaf nodes	from 2 to 20
Max features	from 2 up to the number of channels
Min impurity decrease	from 0.00005 to 0.01

**Table 3:** The table lists the main parameters for configuring a Random Forest model, including criterion, max depth, min samples split, min samples leaf, max leaf nodes, max features, min impurity decrease, and n estimators. These parameters control the quality of splits, the depth of individual trees, and the overall number of trees in the forest, influencing the model’s performance and generalization.

Parameter	Values
Criterion	gini, entropy
N estimators	from 1 up to 100
Min samples split	from 5 up to 100
Min samples leaf	from 5 up to 100
Max leaf nodes	from 2 to 20
Max features	from 2 up to the number of channels −1
Min impurity decrease	from 0.00005 to 0.01
Max depth	From 2 up to 100

**Table 4:** The table lists the main parameters for configuring a K-Nearest Neighbors model, including n neighbors, weights, leaf size, and metric. These parameters determine the number of neighbors to consider, how distances are weighted, the size of the leaf nodes in the search tree, and the distance metric used, affecting the model’s accuracy and performance.

Parameter	Values
N neighbours	From 5 up to 100
Weights	Uniform or distance
Leaf size	from 2 up to 150
Metric	Euclidean, Manhattan, Minkowski or Chebyshev

**Table 5:** The parameters and their possible values for XGBoost are outlined. For any parameters not included in the list, the default values provided by the function were applied.

Parameter	Values
Learning rate	From 0.001 up to 0.3
N estimators	from 2 up to 120
Booster	Gbtree, gblinear or dart
gamma	from 0 up to 6
subsample	From 0 up to 0.9
Col sample by tree	From 0 up to 0.9
Reg alpha	from 0.00005 to 100
Max depth	From 2 up to 20

**Table 6:** The table displays Brodmann Areas (BAs) involved across EEG frequency bands and cognitive control behaviors. Rows represent BAs in ascending numerical order, while columns correspond to EEG bands and behavioral conditions. Cells highlighted in green indicate BA activation in the S condition, orange in the R condition, and purple in the K condition, for the corresponding EEG band and behavior.

	THETA			ALPHA HIGH			BETA1			BETA2			BETA3			GAMMA		
BAs	S	R	K	S	R	K	S	R	K	S	R	K	S	R	K	S	R	K
**1**																		
**2**																		
**3**																		
**4**																		
**5**																		
**6**																		
**7**																		
**8**																		
**9**																		
**10**																		
**11**																		
**12**																		
**13**																		
**14**																		
**15**																		
**16**																		
**17**																		
**18**																		
**19**																		
**20**																		
**21**																		
**22**																		
**23**																		
**24**																		
**25**																		
**26**																		
**27**																		
**28**																		
**29**																		
**30**																		
**31**																		
**32**																		
**33**																		
**34**																		
**35**																		
**36**																		
**37**																		
**38**																		
**39**																		
**40**																		
**41**																		
**42**																		
**43**																		
**44**																		
**45**																		
**46**																		
**47**																		

**Table 7:** The values presented for each subject correspond to their accuracy with a window size of 35 s. Notably, seven out of eleven participants exceed the accuracy threshold of 0.70, demonstrating a strong overall performance of the KNN model across most participants. The subjects with accuracy values exceeding 0.70 are highlighted in green.

Subjects	Accuracy
Subject 1	0.80
Subject 2	0.81
Subject 3	0.85
Subject 4	0.62
Subject 5	0.46
Subject 6	0.75
Subject 7	0.94
Subject 8	0.67
Subject 9	0.74
Subject 10	0.93
Subject 11	0.61

**Table 8:** Metrics for the binary model: Precision 0, Precision 1, Recall 0, Recall 1, Specificity 0, Specificity 1, F1-score 0, F1-score 1, and Accuracy of KNN model. The data presented specifically refer to a window size of 35 s. It is noteworthy that each metric reaches very high values, greater than or equal to the 0.70 threshold. This indicates a robust and reliable model capable of performing well across various evaluation areas, such as precision, recall, and overall accuracy.

Metric	Value
Precision 0	0.73
Precision 1	0.74
Recall 0	0.79
Recall 1	0.71
Specificity 0	0.71
Specificity 1	0.79
F1-score 0	0.70
F1-score 1	0.75
Accuracy	0.74

**Figure A1:**
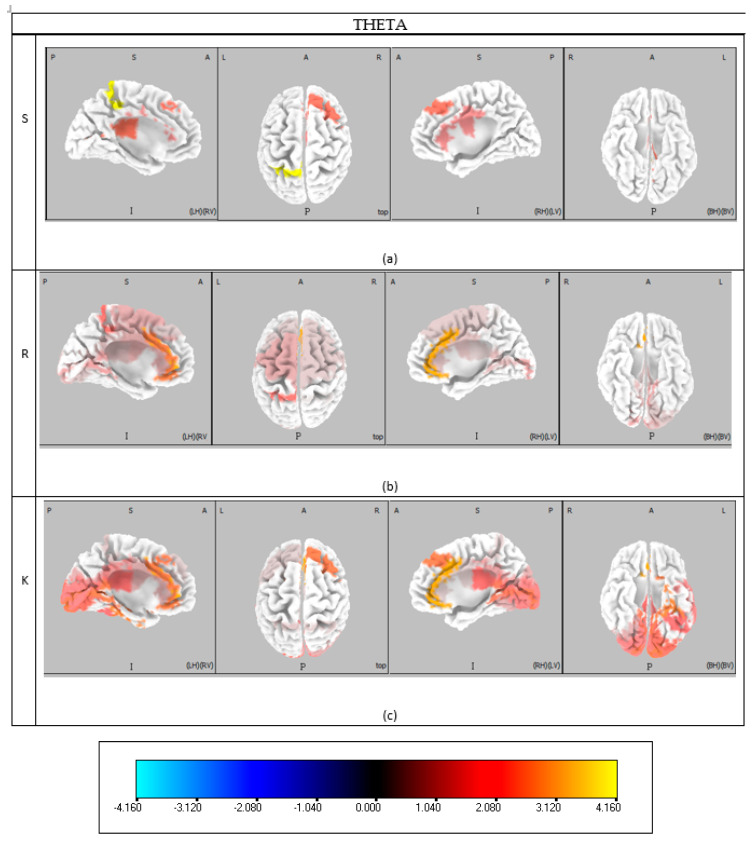
4 Orthogonal views of Theta band in Skill (**a**), Rule (**b**) and Knowledge (**c**) based behaviour. Each figure presents four perspectives: the right hemisphere viewed from the right, from above, from the left, and a perspective from below. Brain anatomy is depicted in grey color. Brain activity increments are in red, no-activations in white, and brain activity decrements in blue colours. Coloured areas represent the t-value, or the differences of the medians. Over each image, there are letters following the following legend: A = anterior, P = posterior, S = superior, I = inferior, L = left, R = right.

**Figure A2:**
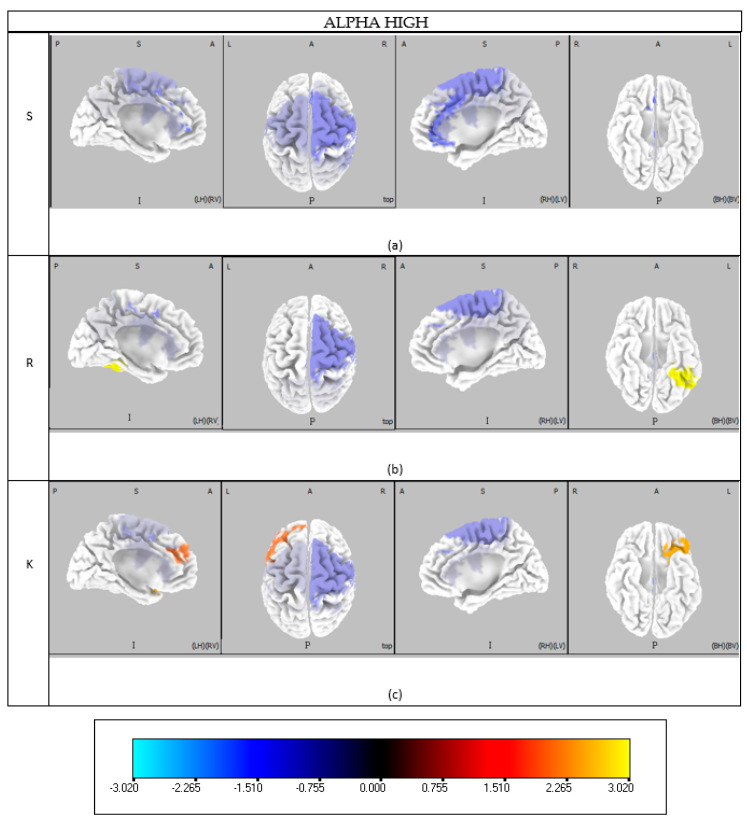
Four orthogonal views of Alpha High band in Skill (**a**), Rule (**b**) and Knowledge (**c**) based behaviour. Each figure presents four perspectives: the right hemisphere viewed from the right, from above, from the left, and a perspective from below. Anatomy is depicted in shades of grey. Positive LORETA values are in red, zero in white, and negative values in blue. Coloured areas represent the t-value, or the differences of the medians. Over each image, there are letters following the following legend: A = anterior, P = posterior, S = superior, I = inferior, L = left, R = right.

**Figure A3:**
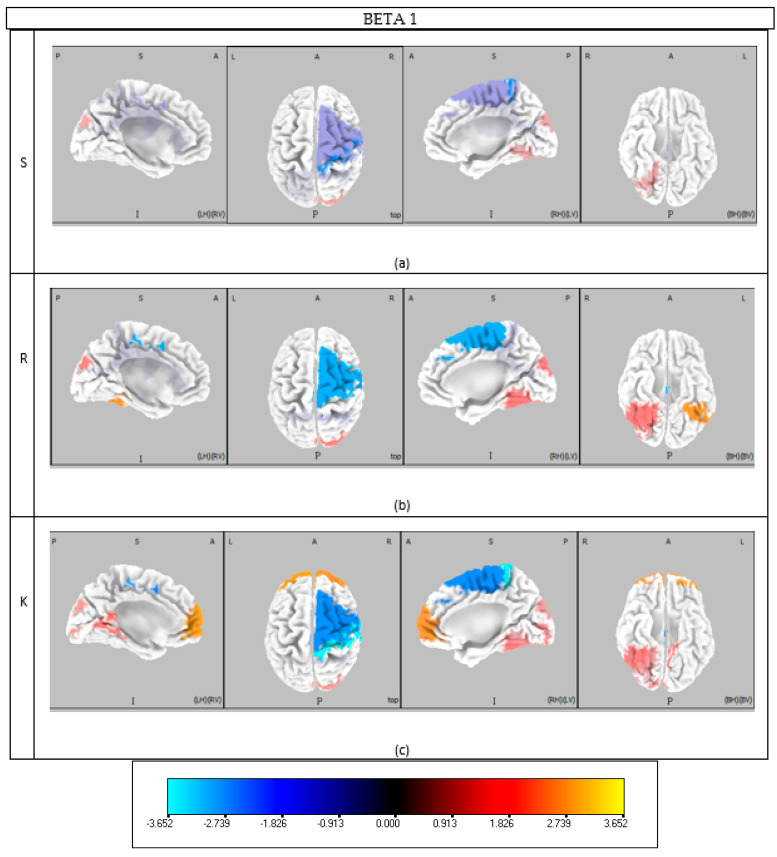
Four orthogonal views of Beta 1 band in Skill (**a**), Rule (**b**) and Knowledge (**c**) based behaviour. Each figure presents four perspectives: the right hemisphere viewed from the right, from above, from the left, and a perspective from below. Anatomy is depicted in shades of grey. Positive LORETA values are in red, zero in white, and negative values in blue. Coloured areas represent the t-value, or the differences of the medians. Over each image, there are letters following the following legend: A = anterior, P = posterior, S = superior, I = inferior, L = left, R = right.

**Figure A4:**
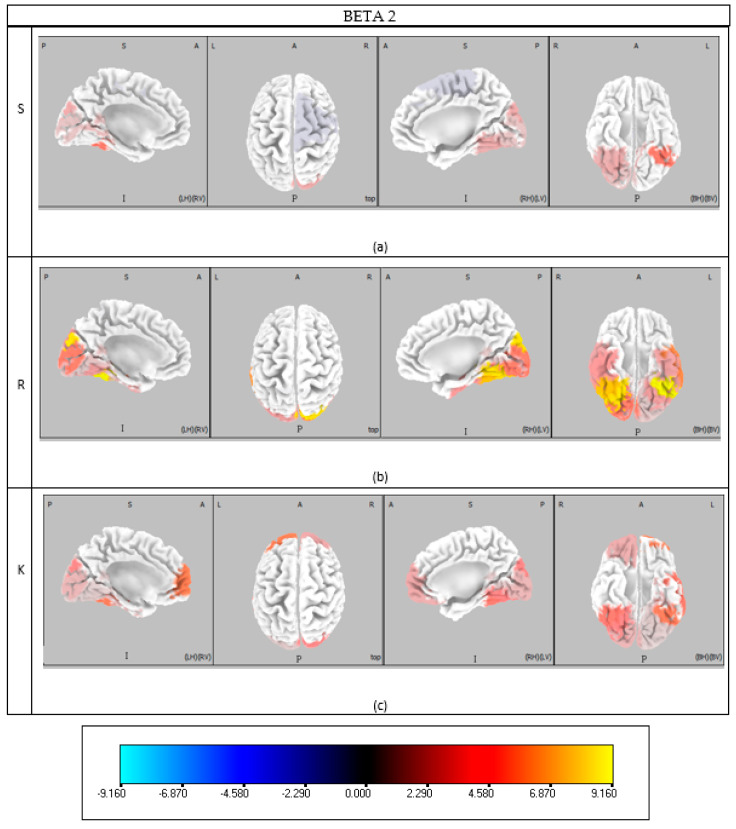
Four orthogonal views of Beta 2 band in Skill (**a**), Rule (**b**) and Knowledge (**c**) based behaviour. Each figure presents four perspectives: the right hemisphere viewed from the right, from above, from the left, and a perspective from below. Anatomy is depicted in shades of grey. Positive LORETA values are in red, zero in white, and negative values in blue. Coloured areas represent the t-value, or the differences of the medians. Over each image, there are letters following the following legend: A = anterior, P = posterior, S = superior, I = inferior, L = left, R = right.

**Figure A5:**
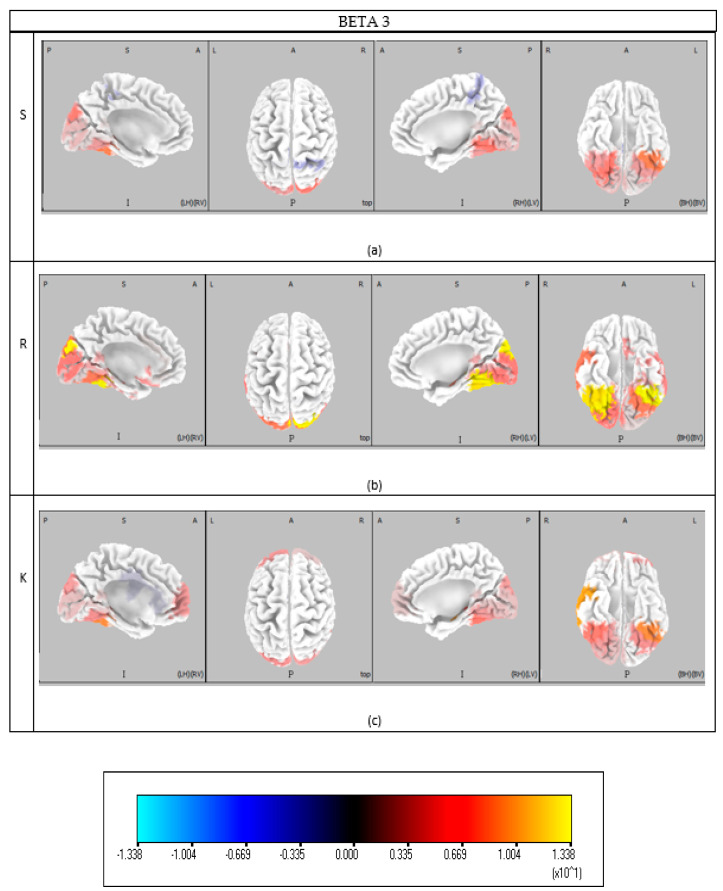
Four orthogonal views of Beta 3 band in Skill (**a**), Rule (**b**) and Knowledge (**c**) based behaviour. Each figure presents four perspectives: the right hemisphere viewed from the right, from above, from the left, and a perspective from below. Anatomy is depicted in shades of grey. Positive LORETA values are in red, zero in white, and negative values in blue. Coloured areas represent the t-value, or the differences of the medians. Over each image, there are letters following the following legend: A = anterior, P = posterior, S = superior, I = inferior, L = left, R = right.

**Figure A6:**
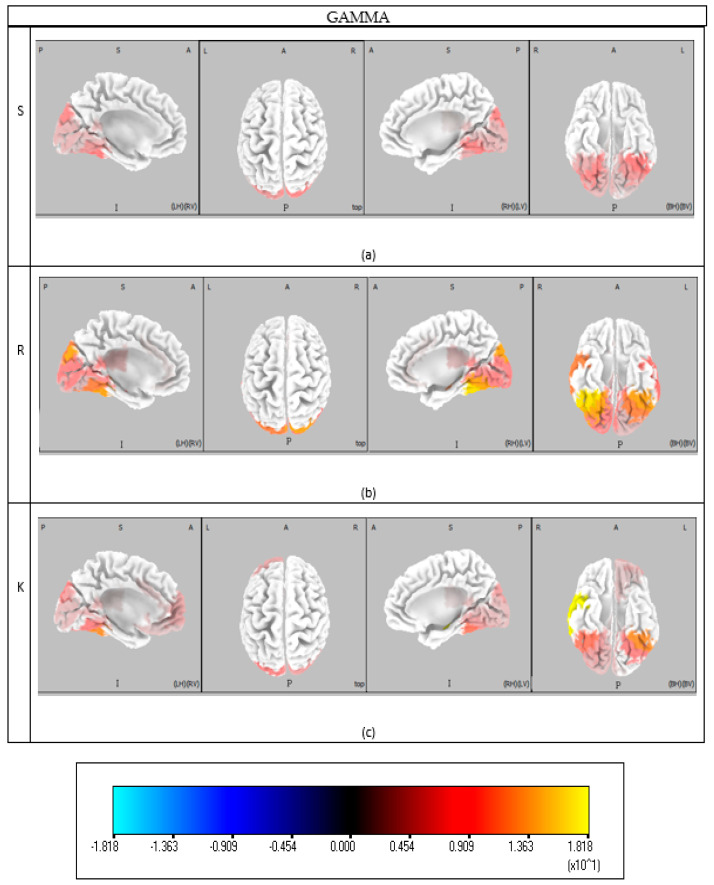
Four orthogonal views of Gamma band in Skill (**a**), Rule (**b**) and Knowledge (**c**) based behaviour. Each figure presents four perspectives: the right hemisphere viewed from the right, from above, from the left, and a perspective from below. Anatomy is depicted in shades of grey. Positive LORETA values are in red, zero in white, and negative values in blue. Coloured areas represent the t-value, or the differences of the medians. Over each image, there are letters following the following legend: A = anterior, P = posterior, S = superior, I = inferior, L = left, R = right.

## Data Availability

The data presented in this study are available on request from the corresponding author due to privacy and ethical reasons.

## Appendix A

**Table A1:** Per-subject performance metrics for the binary classification (Knowledge vs. Skill + Rule). Metrics include Precision, Recall, Specificity, F1-score, and Accuracy for each class, reported as mean ± 95% confidence interval across cross-validation folds. Confusion matrix entries (TN, FP, FN, TP) are also provided for each subject.

Subjects	Precision 0	Precision 1	Recall 0	Recall 1	Specificity 0	Specificity 1	F1-Score 0	F1-Score 1	Accuracy	TN	FP	FN	TP
Subject 1	0.79 ± 0.12	0.81 ± 0.06	0.35 ± 0.07	0.98 ± 0.01	0.98 ± 0.03	0.35 ± 0.03	0.74 ± 0.13	0.88 ± 0.05	0.80 ± 0.05	15	28.5	4	159
Subject 2	0.67 ± 0.10	0.83 ± 0.06	0.63 ± 0.11	0.86 ± 0.06	0.86 ± 0.08	0.63 ± 0.07	0.65 ± 0.11	0.84 ± 0.06	0.81 ± 0.05	48	28.5	24.5	211
Subject 3	0.90 ± 0.04	0.87 ± 0.05	0.84 ± 0.05	0.50 ± 0.00	0.50 ± 0.00	0.84 ± 0.03	0.85 ± 0.07	0.93 ± 0.04	0.85 ± 0.06	75.5	15.5	11	100.5
Subject 4	0.63 ± 0.06	0.68 ± 0.09	0.54 ± 0.09	0.88 ± 0.02	0.88 ± 0.05	0.54 ± 0.08	0.36 ± 0.12	0.71 ± 0.10	0.62 ± 0.07	31.5	28.5	75.5	258.5
Subject 5	0.63 ± 0.06	0.24 ± 0.32	0.80 ± 0.06	0.50 ± 0.00	0.50 ± 0.00	0.80 ± 0.18	0.59 ± 0.13	0.39 ± 0.36	0.46 ± 0.09	41.5	11	68	3.5
Subject 6	0.77 ± 0.05	0.76 ± 0.07	0.73 ± 0.05	0.90 ± 0.02	0.90 ± 0.04	0.73 ± 0.07	0.66 ± 0.10	0.78 ± 0.08	0.75 ± 0.06	61.5	23.5	36	166.5
Subject 7	0.93 ± 0.05	0.98 ± 0.02	0.96 ± 0.03	0.89 ± 0.07	0.89 ± 0.06	0.96 ± 0.02	0.94 ± 0.06	0.93 ± 0.07	0.94 ± 0.05	49.5	2.5	3.5	73
Subject 8	0.67 ± 0.10	-	1.00 ± 0.00	0.00 ± 0.00	0.00 ± 0.00	1.00 ± 0.00	0.78 ± 0.09	-	0.67 ± 0.07	66	0	44	0
Subject 9	0.66 ± 0.06	0.73 ± 0.22	0.96 ± 0.03	0.72 ± 0.03	0.72 ± 0.06	0.96 ± 0.12	0.72 ± 0.08	0.62 ± 0.24	0.74 ± 0.06	66.5	3	65.5	53.5
Subject 10	0.84 ± 0.05	0.79 ± 0.17	0.95 ± 0.05	0.95 ± 0.01	0.95 ± 0.03	0.95 ± 0.09	0.88 ± 0.06	0.85 ± 0.15	0.93 ± 0.03	89	5	19.5	183
Subject 11	0.57 ± 0.04	0.70 ± 0.16	0.90 ± 0.05	0.67 ± 0.02	0.67 ± 0.05	0.90 ± 0.13	0.57 ± 0.08	0.53 ± 0.18	0.61 ± 0.06	66.5	7	230.5	118
